# C-fiber-related brain responses evoked by laser heat pulses applied to the back

**DOI:** 10.1016/j.jphyss.2025.100018

**Published:** 2025-03-24

**Authors:** Benjamin Provencher, Mathieu Piché

**Affiliations:** Department of Anatomy, Université du Québec à Trois-Rivières, Trois-Rivières, QC G9A 5H7, Canada

**Keywords:** C-fibers, Lasers, Evoked-potentials, Back Pain, Brain activity

## Abstract

The aim of the present study was to examine C-fiber-related brain responses evoked by laser heat stimuli applied to the lumbar area, and to determine the stimulation protocol that produces the most reliable responses. Thirty healthy volunteers completed the study. Combinations of different stimuli (single pulses or trains of three pulses) with different pulse durations (7 or 14 ms) were used to compare C-fiber-related brain responses between protocols**.** The four protocols elicited comparable C-fiber-related brain responses to laser heat pulses. However, pulse trains of 7 ms pulses at 0.67 Hz elicited C-LEPs in the greatest proportion of participants (86.7 %). C-LEPs occurred within a 500 ms to 1500 ms post-stimulus time window, consistent with the perception associated with C-fiber activation. These results provide novel data on C-fiber-related brain responses to painful stimuli and a reliable stimulation protocol for future studies on low back pain.

## Highlights

• Laser pulse trains applied to the back produce temporal summation of second pain.
• These stimuli also allow the detection of C-fiber brain responses in most individuals.
• This provides a reliable stimulation protocol for future studies on low back pain.

## 1 Introduction

Laser-evoked brain responses constitute an essential tool to investigate the nociceptive system, pain-related processes, and their regulation [1], [2], [3], [4], [5], [6], [7]. Laser heat pulses activate Aδ and C fibers selectively [1], [8], avoiding the confound of and the interaction with touch-related activation of Aβ fibers. Nociceptive C fibers are of particular interest for the study of nociplastic pain. They are involved in windup and central sensitization and are thought to contribute to secondary hyperalgesia and allodynia [9], [10], [11].

The repeated activation of C-fiber afferents at relatively short intervals leads to windup and its perceptual correlate, temporal summation of pain [12], [13], [14], while the activation of C-fiber afferents caused by injuries contribute to central sensitization and chronic pain [10], [15], [16], [17]. Although windup and central sensitization represent distinct processes, both rely on C-fiber activity [9], [18], [19] and both are relevant to the investigation of acute and chronic pain mechanisms.

The measurement of brain responses related to C-fiber activation remains challenging [1], [2], [3], [8]. This may explain the wide variety methods reported previously, including laser-evoked potentials (LEP) (see Table S1 in the supplementary material). The “first come first serve” hypothesis has been proposed, where “only the earliest of a series of somatosensory volleys elicits cerebral responses synchronous enough to yield EPs [evoked potentials]” [20]. This hypothesis is based on the observation that C-LEPs could be detected only if Aδ fiber activation was limited by various procedures [21], [22], [23], [24], [25], [26]. It is also supported by empirical evidence showing that brain activity related to Aδ fiber activation is inhibited by preceding C-fiber-related responses [27]. However, the hypothesis was challenged by the demonstration of clear Aδ-LEPs observable even when preceded by Aβ afferent volleys [28]. Despite this report, current studies on C-LEPs include procedures to limit the activation of Aδ fibers [29], [30], [31], [32], [33], [34], [35]. These methods are not adapted to all research protocols and often lead to perceptual ratings below the pain threshold [33], [34], [36], [37], [38], [39], [40]. This is a major drawback for pain studies. It was proposed that C-LEPs induced by laser heat stimulation of the hand can be detected in most participants when using adapted stimulation parameters [41]. However, the results could not be replicated for back stimulation [5], [6]. In the present study, the lower back was favored over the hand dorsum to apply the results to future studies investigating low back pain.

The aim of the present study was to establish a protocol to investigate brain activity evoked by the activation of C fibers in the lumbar area. Based on previous studies (see Table S1 in the supplementary material), single or repeated laser pulses (3) were used, with a pulse duration of 7 or 14 ms. We hypothesized that laser pulse trains would elicit measurable C-fiber-related responses in a greater proportion of participants compared with single pulses. Moreover, we expected that laser pulse trains at 0.67 Hz would elicit an increase in the amplitude of C-fiber-related brain responses between the first and last train pulse.

## 2 Methods

### 2.1 Sample size calculation

The sample size was determined using G*Power 3.1.9.7 software [42]. According to our hypothesis and experimental design, the sample size was established to detect a medium effect size (η_p_^2^ = 0.06) for the interaction between the stimulus type (single pulse vs. trains) and stimulus duration (7 ms vs. 14 ms), with a power of 0.8 and an α of 0.05. The calculation resulted in a sample size of 34, which was rounded up to 40 participants to account for potential exclusions.

### 2.2 Participants

**Fig. 1:**
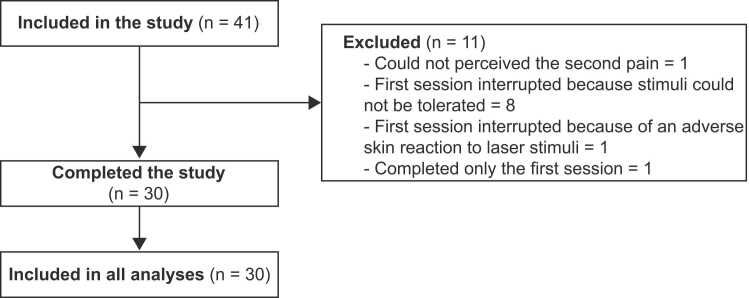
Flow chart of participants included in the study and analyses. Forty-one healthy volunteers met the inclusion criteria and were included in the study. Eleven participants were excluded after inclusion, resulting in thirty participants that completed the study (14 women and 16 men; mean age ± SD: 30.9 ± 7.9 years). Participants that were excluded from the study (n = 11/41) did not complete the experiment so their data could not be analyzed. On these 11 participants, one could not perceive second pain, one presented an adverse skin reaction to the laser stimuli, eight could not tolerate the laser pulse trains, and one completed only one session. Among the thirty participants that were included, all data was of good quality and no participant was excluded during the analyses.

Healthy volunteers were recruited by advertisements on the campus of Université du Québec à Trois-Rivières and on social media. They were included if they were between 18 and 55 years old and in good general health. They were excluded if they reported acute or chronic pain, acute or chronic illness, psychiatric disorders or daily usage of recreational drugs, if they had an injury or surgery in the 3 months preceding the experiment, and if they took any pain medication or recreational drug within 48 h prior to the experimental session. They were also excluded if they presented a skin of type I on the Fitzpatrick scale [43], if they could not perceive the second pain evoked by laser stimuli, or presented an adverse skin reaction to laser stimulation. Forty-one healthy volunteers met the inclusion criteria and were included in the study. Eleven participants were excluded after inclusion, resulting in thirty participants that completed the study (14 women and 16 men; mean age ± SD: 30.9 ± 7.9 years). Participants that were excluded from the study (n = 11/41) did not complete the experiment so their data could not be analyzed (see Fig. 1). On these 11 participants, one could not perceive second pain, one presented an adverse skin reaction to the laser stimuli, eight could not tolerate the laser pulse trains, and one completed only one session. Among the thirty participants that were included, all data was of good quality and no participant was excluded during the analyses.

### 2.3 Experimental design and protocol

This study relies on a within-subject design to compare C-fiber responses between four stimulation protocols (single pulses vs. pulse trains of 7-ms or 14-ms duration). During the experiment, room temperature was kept constant at 24°C. All participants were seated on a regular desk chair. A 22×14 cm opening in the center of the backrest exposed the skin of the lower back for stimulation. The participant and experimenter were wearing safety glasses designed for a 1340 nm wavelength laser during the experiment. Participants were instructed to keep their eyes open, look at a fixation cross to minimize eye movement, and refrain movement as much as possible during the recordings.

**Fig. 2:**
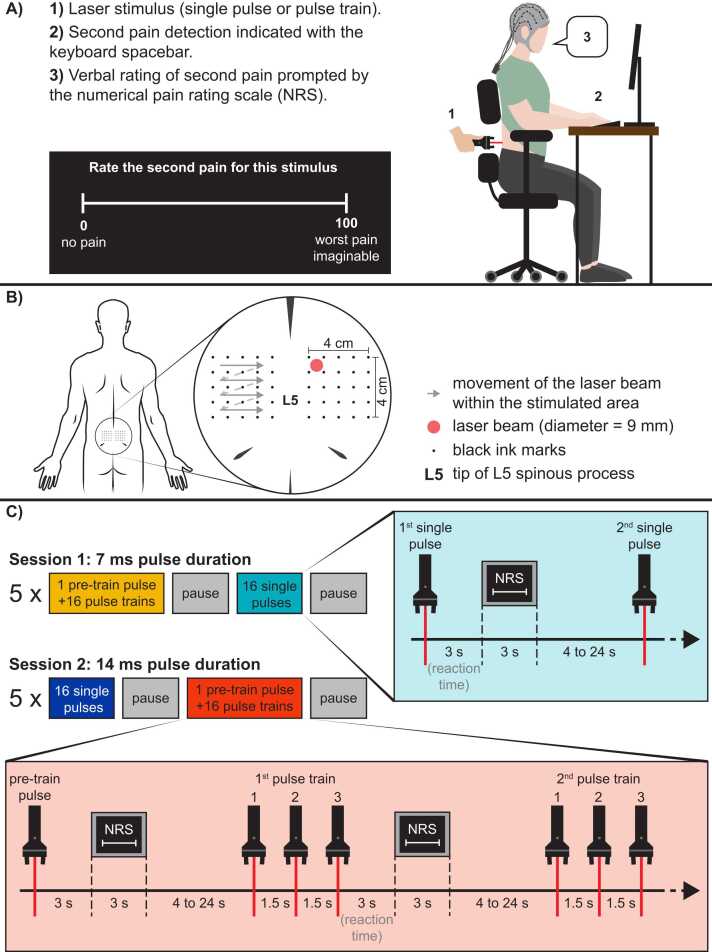
Experimental protocol. Detailed representation of the experimental setup, stimulation procedures and protocol. A) The laser stimulus was delivered to the back area. Participants indicated the detection of second pain by pressing the spacebar. Reaction times were extracted to calculate the second pain perception occurrence. After each stimulus, participants were prompted to rate second pain with the display of a numerical pain rating scale. B) Illustration of skin landmarks (grid of ink marks) drawn around L5 vertebrae for the application of laser stimuli bilaterally. The 9 mm laser beam was carefully positioned at one of the 32 spots within this stimulation grid and on ink-free skin. The laser beam was moved to the next spot in the grid after each stimulus (single pulse, pre-train pulse, and pulse train) for safety purposes. It was moved sequentially from left to right and top to bottom. Within a pulse train however, the three pulses were delivered to the same spot to induce temporal summation, within safety limits. C) Stimulation protocol. Participants came to the laboratory for two experimental sessions. In both sessions, they received 5 series of stimuli, including 16 pulse trains preceded by one pre-train pulse for comparison, to assess temporal summation, and 16 single-pulse stimuli. One session comprised 7 ms stimuli while the other comprised 14 ms stimuli. Only one sequence is illustrated here, but the order of the sessions (7 ms or 14 ms) and the order of stimulus presentation (single pulse or pulse train) was counterbalanced between participants. Numbers (1, 2 and 3) represent the first, second and third pulse of the pulse trains. NRS: Numerical Rating Scale.

The experimental protocol is illustrated in Fig. 2. Participants took part in two experimental sessions separated by approximately one week (mean ± SD: 8.3 ± 3.5 days). The two sessions were identical except for the laser pulse duration that was either 7 ms or 14 ms. The order of the sessions was counterbalanced between participants. Each session comprised two stimulation types: single pulses and pulse trains (three pulses at a frequency of 0.67 Hz). During each session, a total of 165 stimuli were delivered on either side of the L5 spinous process, including five blocks of 16 single-pulse stimuli (n = 80), five blocks of 16 pulse-train stimuli (n = 80) and five pre-train pulse (see Fig. 2C). The number of stimuli was determined based on the results of a previous study indicating that 80 stimuli provide a good signal-to-noise ratio for C-LEPs [41]. In the present experiment, the 80 stimuli were grouped in blocks of 16 stimuli to limit habituation, based on results from two of our previous studies indicating that significant habituation of C-fiber responses occurred after 15–20 stimuli [5], [6]. The order of stimuli (single-pulse or pulse-train stimuli) was counterbalanced between participants and sessions. A pause followed each stimulation block to allow participants to move. The pause was not time-limited but lasted less than 2 min. Reaction times and pain ratings were measured as described below, during continuous electroencephalographic recording (EEG).

### 2.4 Pain ratings

Three seconds after each stimulus, participants were prompted to rate second pain verbally by a numerical scale (NRS) that appeared on the computer screen, where 0 indicated “no pain” and 100 indicated the “worse pain imaginable” [5]. To avoid contaminating the EEG signals, when the stimulus was a pulse train, participants were instructed to rate the third pulse only. As illustrated in Fig. 2C, during the experiment, a pre-train pulse of the same stimulus intensity was delivered before each pulse train stimulation block. The rationale behind these pre-train pulses was to use their pain ratings to quantify the temporal summation of second pain induced by pulse trains. They were only used for the pain rating analysis to confirm temporal summation of second pain.

### 2.5 Painful laser stimulation

Painful stimuli were produced by laser heat pulses generated by an infrared neodymium-doped yttrium aluminum perovskite laser (Nd:YAP, DEKA 1340, Electronical Engineering, Florence, Italy). Infrared laser stimuli have been shown to activate heat sensitive Aδ and C fibers selectively, while avoiding the activation of Aβ fibers [8], [44]. The laser beam was transmitted through a 10 m optic fiber cable and was adjusted to a diameter of 9 mm (≈63.6 mm^2^ area), with a pulse duration of 7 or 14 ms. These pulse durations were selected after pilot testing. With these parameters, stimuli could be adjusted within a broad range of intensity (1.5 to 8.5 J) to induce pain perception that was tolerable and comparable between sessions for all participants. Similar pulse durations have been used to measure C-LEPs in previous studies, although parameters vary between experiments and laboratories (see Table S1 in the supplementary material).

The interstimulus interval varied randomly from 10 to 30 s to limit predictability and habituation [45], [46], [47]. Pulse trains consisted of 3 pulses repeated at 0.67 Hz, which induced temporal summation of second pain [12], [13], [18] while allowing the detection of C-fiber-related brain responses for each pulse of the train. Indeed, C-LEP peaks have been measured within a 1500 ms window post-stimulus in previous studies [31], [36], [37], [41]. The maximal fluence used during the present study was 134 mJ/mm^2^ for single pulses and 51 mJ/mm^2^ for each pulse of the pulse trains. The laser was triggered using a stimulus presentation software (E-Prime2; Psychology Software Tools, Inc., Pittsburgh, PA, USA). To avoid stimulating the same area too frequently, 25 tiny ink marks were drawn on the skin with a marker (Staedtler triplus® fineliner 334, color: black, line width: 0.3 mm) to guide the experimenter. The marks were made within two 4 × 4 cm grids, one on each side of L5 spinous process. Stimulating blackened skin with a Nd:YAP laser results in higher temperature than stimulating unmarked skin, which increases the probability of reaching Aδ fiber threshold [48]. Therefore, during the present experiment, the laser beam was aimed at the space between the marks and it was moved sequentially to the next space in the grid after each stimulus (see Fig. 2). The movement of the laser beam within the grids was kept the same across participants and sessions.

At the beginning of each session, pain thresholds and laser intensity evoking low to moderate pain were determined using a staircase procedure [6], [49]. This was done separately for each stimulus type (single pulse and pulse trains), because they induce pain of different intensity. Before pain threshold assessment, the experimenter explained the concept of second pain to the participant with visual support using a self-made figure. Participants were then instructed to focus on the second pain (the dull/hot/burning sensation in their back) and to report its intensity verbally after each stimulus, using a numerical rating scale ranging from 0 to 100, 0 indicating “no pain” and 100 “the worse pain imaginable.” Stimuli were delivered at an initial intensity of 1.5 J and stimulus intensity increased sequentially by 0.25 J increments until pain was reported (rating of 1/100 or higher). Then, the energy was increased sequentially again until a pain rating above 30/100 was reported. Participants were then familiarized with the selected intensity using three to five consecutive stimuli with an interstimulus interval varying between 5 and 10 s. If the intensity was deemed acceptable for the participant, the experiment continued. If the participant judged that the stimulus intensity produced pain that could not be tolerated for the duration of the experiment, stimulus intensity was decreased by 0.25 J and the familiarization procedure resumed until an acceptable stimulus intensity was reached. The average laser stimulation intensity was 5.7 ± 1.5 J (7 ms – single pulse), 5.5 ± 1.5 J (14 ms – single pulse), 2.2 ± 0.6 J (7 ms – pulse train) and 2.1 ± 0.5 J (14 ms – pulse train). The skin was visually monitored throughout the entire session. One participant presented an adverse skin reaction to laser stimuli. The experiment was stopped as soon as it was noticed, and the follow-up revealed that the skin healed completely within a few weeks. No other adverse event was observed by the experimenter or was reported by participants. After each session, the lumbar region was inspected. No swelling, redness, or other signs of inflammation or damage were noted.

Before the experiment, participants completed at least 10 practice trials (5 single pulses and 5 pulse trains) for familiarization with the procedures. During these trials, their reaction times (see Section 2.6) were displayed on a screen and used to determine that participants understood the instructions and were able to perceive second pain. Participants were excluded from the study if they could not perceive the second pain, as assessed by the reaction times during the practice trials (latencies <650 ms, corresponding to Aδ-fiber activation). This was necessary since the present study aimed to measure pain-related brain responses evoked by nociceptive C-fibers. Only one participant was excluded for this reason.

### 2.6 Reaction times

Participants were instructed to press the keyboard spacebar as soon as they perceived the second pain caused by a painful laser stimulus, as described previously [50]. When the stimulus was a pulse train, participants were instructed to press as soon as they perceived the second pain caused by the third pulse. Reaction times were recorded by the stimulus presentation software that triggered the laser (see above). Based on previous studies, reaction times greater than 650 ms were considered consistent with the perception of a C-fibers mediated sensation [30], [31], [33], [36], [37], [38], [51], [52].

### 2.7 Electroencephalographic recordings

EEG was recorded using a 64-channel BrainVision system with active Ag-AgCl electrodes mounted on an actiCAP, according to the International 10–20 system (Brain Products, Gilching, Germany). Electrodes were nose-referenced, and the ground was set at FPz [53], [54], [55]. Eye movements and blinks were recorded using electrooculography (EOG) with electrodes placed at the right suborbital ridge, and just lateral to the right external ocular canthus. Electrode impedance was kept below 20 kΩ. Signals were sampled at 1000 Hz and filtered using a 0.01–100 Hz band-pass filter.

### 2.8 Laser-evoked potentials

EEG signals were analyzed offline using MATLAB R2022a (MathWorks, Natick, MA, USA) and EEGLAB v2022.0 [56]. EEG signals were preprocessed using a standardized, automated, and open-source preprocessing pipeline for EEG data [57]. The preprocessing steps are briefly described here: 1- Downsampling the data from 1000 Hz to 500 Hz; 2- Re-referencing signals to the common average; 3- Automatic detection and spherical interpolation of channels of low quality using the outlier criterion z > 3.29 [58]; 4- Segmenting the data into “long” epochs (−2000 ms to 7000 ms) to get better independent component analysis (ICA) solution in the next steps; 5- High-pass filtering using a 0.5 Hz finite impulse response (FIR) filter; 6- First ICA using the built-in function Runica in EEGLAB; 7- Automatic detection and rejection of contaminated trials based on z-value detection (> 3.29) of independent components (ICs); 8- Second ICA; 9- Automatic detection and rejection of the ICs related to noise using the ICLabel plugin [59] with preset thresholds (> 90 % for muscles and eyes, > 95 % for heart, > 75 % for line and channel noise); 10- Segmenting the data into suitable epochs for analysis (−500 ms to 1500 ms for single pulse conditions and −500 ms to 4500 ms for pulse train conditions); 11- Baseline correction (−500 ms to 0 ms)[31], [36], [37], [60]; 12- Low-pass filtering using a 30 Hz FIR filter. On average, 6.7 ± 1.8 channels were interpolated, 1.3 ± 0.9 epochs were rejected and 85.3 ± 8.0 % of the variance remained after IC rejection.

After data preprocessing, average waveforms were computed for each participant and condition, and the positive slow wave evoked by laser stimuli was analyzed. The peak amplitude and latency of this positive slow wave were measured automatically using the max function within a specified time window on each average waveform. The peak was defined as the maximum value between 500 and 1450 ms with a maximum amplitude at Pz. This time window is based on previous studies measuring C-LEPs [29], [41], [61].

In addition, the proportion of participants presenting C-LEPs was calculated for each condition. These proportions were obtained by the two authors agreeing on the presence or absence of C-LEPs based on individual LEP waveforms and topographic maps.

### 2.9 Event-related spectral perturbation analyses

Event-related spectral perturbations (ERSP) were examined based on recent studies reporting that they present several advantages over LEPs to measure EEG activity that is not time-locked or is subject to latency jitters [2], [62]. In order to measure ERSP, EEG datasets were preprocessed using the same automated preprocessing pipeline as described above, with different values for filters (High-pass: 1 Hz; Low-pass: 100 Hz), baseline (−700 ms to −200 ms) and the final length of the epochs (−2000 ms to 2000 ms for single pulse conditions and −2000 ms to 5000 ms for pulse train conditions). Then, as in previous studies [6], [55], a Morlet wavelet convolution [63] was computed using the channel time–frequency options available in EEGLAB v.13.5.4b [56]. Four hundred or 1200 time points were generated (single pulse or pulse train conditions), and 100 linearly spaced frequencies were computed from 1 to 100 Hz. Variable cycles were used for low and high frequencies, with 3 cycles for lowest frequencies and up to 15 cycles for highest frequencies [56]. This variable number of cycles allows for the wavelet convolution method to provide a better frequency resolution at lower frequencies and a better temporal resolution at higher frequencies [64]. Time-frequency analyses were performed using time–frequency options available in EEGLAB which use Morlet wavelets. A Gaussian shape taper was used to taper wavelets to zero at both ends, and the length of the window used for time-frequency analysis was selected to avoid the contamination of the signal of interest by edge artifacts. ERSP data were computed in decibels relative to baseline for all electrodes separately. The time–frequency data of all trials were averaged for each participant and condition separately, resulting in 4 average time–frequency maps for each electrode. From these maps, the mean power was extracted from the Cz electrode in predetermined time × frequency regions of interests (ROIs) for each participant. Then, a mean ERSP value was then obtained for each participant and ROI by selecting and averaging the values with the 20 % highest or lowest amplitude (for power increase or decrease relative to baseline).

Since the present study is the first to examine brain responses evoked by laser pulse trains, C-fiber conduction velocity (0.5 - 2 m/s)[65], visual inspection of the time-frequency maps, and results from previous studies [31], [41], [52] were used to define three ROIs at latencies corresponding to C-fiber-related activity. ROI 1 included event-related synchronization (ERS) ranging between 2 and 5 Hz from 500 ms to 1200 ms [31], [41], [52]. Note that these frequencies are within the delta and theta range [66]. ROI 2 included event-related desynchronization (ERD) ranging between 7 Hz to 15 Hz from 1000 ms to 1500 ms [31], [52]. These frequencies are within the alpha range [66]. This ERD was reported to last longer, but the time window is limited to 1500 ms by the pulse train frequency in the present study. ROI 3 included the ERS ranging between 60 and 100 Hz from 500 ms to 1200 ms that was observed in the average time-frequency maps. These frequencies are within the gamma range [66].

### 2.10 Statistical analyses

Statistical analyses were performed with IBM SPSS Statistics for Windows, Version 28.0 (Armonk, NY, USA: IBM Corp). All data are expressed as mean ± SD. Statistical significance threshold was set at p ≤ 0.05. Data distribution was assessed for normality with the Kolmogorov-Smirnov test. Pain ratings and C-fiber-related brain responses were compared between the 7-ms and the 14-ms single-pulse conditions using paired sample *t*-tests. Reaction times were compared between the four conditions using Greenhouse-Geisser corrected repeated-measures ANOVA (2 pulse duration × 2 types of stimuli [single pulse or pulse train]). Temporal summation of second pain was assessed between the pulse train conditions using Greenhouse-Geisser corrected repeated-measures ANOVA (2 pulse durations × 2 types of stimuli [pre-train pulse or pulse train]). C-fiber-related brain responses induced by temporal summation were compared between the pulse train conditions using Greenhouse-Geisser corrected repeated-measures ANOVA (2 pulse durations × 3 pulses [pulse 1, 2 and 3 of each train]). Tuckey HSD tests were used to decompose significant effects. Effect sizes are reported based on partial eta-squared (*η*_*p*_^2^).

## 3 Results

### 3.1 Ratings of second pain

**Fig. 3:**
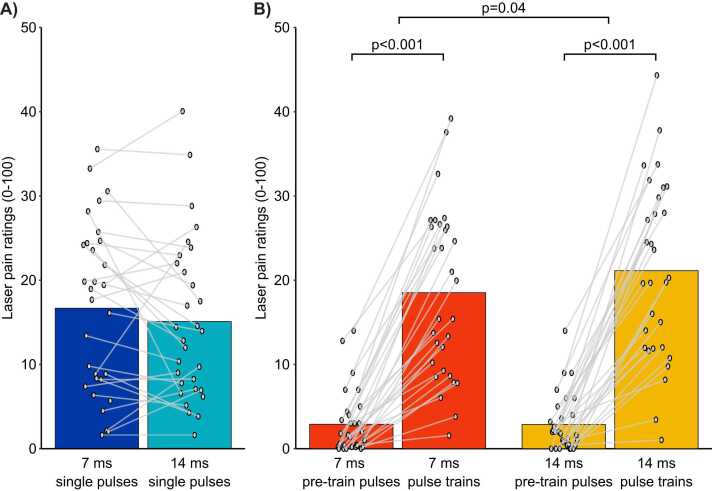
Ratings of second pain and temporal summation of second pain. Data from each participant are represented by linked gray dots and the mean of these data points for each condition is represented by colored bars. A) For single pulses, pain ratings were not significantly different between the 7 ms and 14 ms conditions (p = 0.23). B) For pulse train stimuli, pain ratings were significantly greater compared with the pre-train pulses (p < 0.001), consistent with temporal summation of second pain. Moreover, this effect was significantly greater for the 14 ms compared with the 7 ms pulse duration (p = 0.04).

Ratings of the second pain were compared separately for single pulses and pulse trains. For single pulses, pain ratings were compared between the 7 ms and 14 ms conditions (see Fig. 3A). The paired sample *t*-test revealed no significant difference between conditions (*t*(29) = 1.5, p = 0.2, η_p_^2^ = 0.05), indicating that the pulse duration did not affect pain perception significantly. For pulse trains, pain ratings were compared between the pre-train pulses and the pulse trains for the 7 ms and 14 ms conditions using a two-way repeated-measures ANOVA (2 pulse durations × 2 types of stimuli [pre-train pulse or pulse train (pulse repetition)], see Fig. 3B). Pain ratings were not significantly different between the 7 ms and the 14 ms conditions (main effect of pulse duration: F(1,29) = 1.7, p = 0.2, η_p_^2^ = 0.05). Pain ratings were significantly greater for the pulse trains compared with the pre-train pulses (main effect of pulse repetition: F(1,29) = 129.3, p < 0.001, η_p_^2^ = 0.82), and this effect was significantly different between the 7 ms and the 14 ms conditions (interaction between pulse duration and pulse repetition: F(1,29) = 4.7, p = 0.04, η_p_^2^ = 0.14), where second pain produced by repeated pulses was significantly greater in the 14 ms condition compared with the 7 ms condition.

### 3.2 Reaction times

**Fig. 4:**
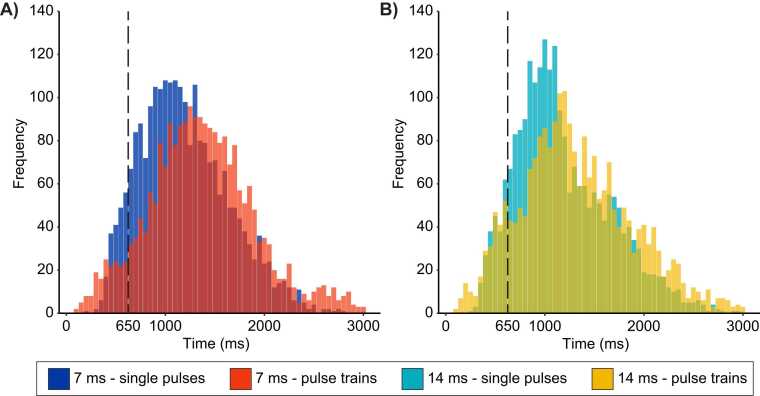
Frequency distribution histograms of trial-by-trial reaction times. Frequency distribution of trial-by-trial reaction times for each condition. The reaction times of trials on the right of the vertical dashed line were equal or greater than 650 ms (89.6 % of trials). Reaction times were significantly longer for pulse trains compared with single pulses (p = 0.002). However, this effect was not significantly different between the 7 ms and the 14 ms conditions (p = 0.90).

Frequency distributions of reaction times (all trials from all participants) for the 7 ms and 14 ms conditions are presented in Fig. 4A-B, with an overlay of distributions for single pulses and pulse trains. A proportion of 89.6 % of the reaction times were ultra-late (greater than 650 ms), consistent with nociceptive C-fiber activation. An additional 3.4 % of the reaction times were missing (no button press), leaving only 7 % of all trials with reaction times < 650 ms. The inclusion of these trials did not have a significant effect on LEP waveforms. Thus, all trials were included in the analysis. The mean reaction time for each condition was 1194 ± 447 ms (7 ms – single pulses), 1364 ± 534 ms (7 ms – pulse trains), 1183 ± 478 ms (14 ms – single pulses), and 1313 ± 563 ms (14 ms – pulse trains). These results are consistent with pain perception related to nociceptive C-fiber activation [33], [36], [37], [51].

Mean reaction times were compared between the 4 conditions using a two-way repeated-measures ANOVA (2 pulse durations × 2 types of stimuli [single pulse or pulse train (pulse repetition)]). The mean reaction times were not significantly different between the 7 ms and the 14 ms conditions (main effect of pulse duration: F(1,29) = 0.3, p = 0.5, η_p_^2^ = 0.01). However, they were significantly longer for pulse trains compared with single pulses (main effect of pulse repetition: F(1,29) = 12.0, p = 0.002, η_p_^2^ = 0.29). This effect was not significantly different between the 7 ms and the 14 ms conditions (interaction between pulse duration and pulse repetition: F(1,29) = 0.02, p = 0.90, η_p_^2^ = 0.01).

### 3.3 Laser-evoked potentials

**Fig. 5:**
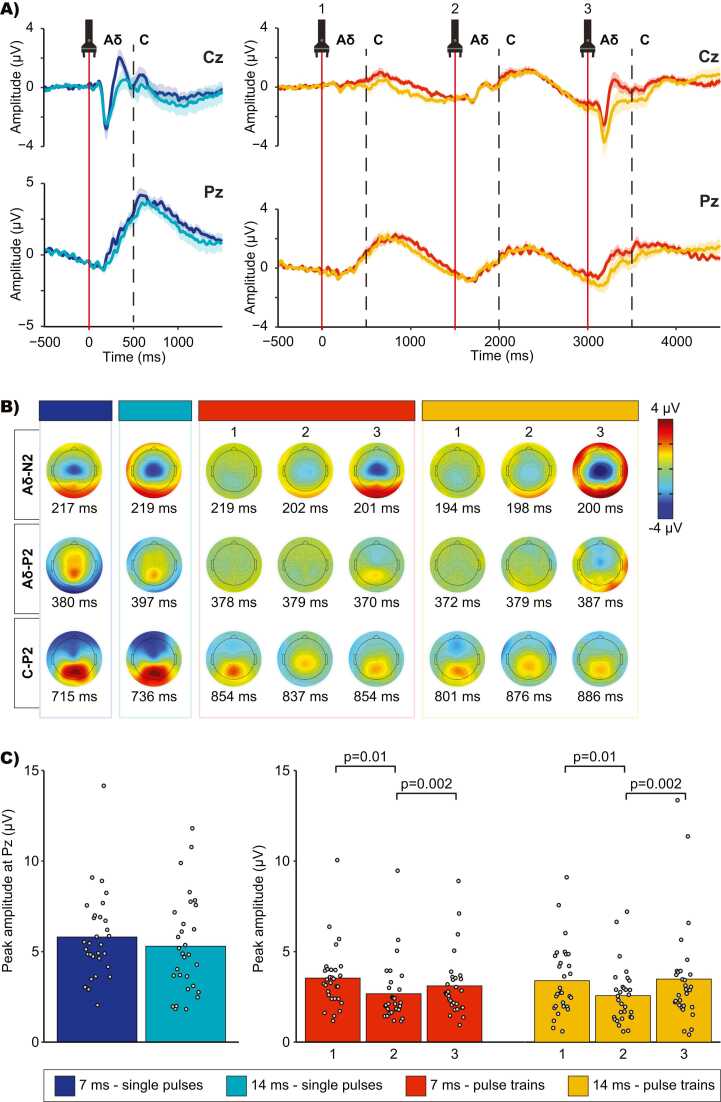
Average LEP waveforms, topographic maps and C-P2 peak amplitudes for each condition. A) Average LEP waveforms for each condition at Cz and Pz. The lightly colored areas above and below the LEP waveforms represent the standard error of the mean (SEM). Both Aδ and C-fibers responses can be visualized at Cz. A vertical dashed line was added 500 ms after each pulse to mark the end of the Aδ-fibers response. At Pz, a slow wave was observed between 500 ms and 1500 ms in every condition, corresponding to the range of C-fiber conduction velocity, and consistent with the reaction times. B) Average topographic maps of the Aδ-N2, Aδ-P2 and C-P2 components for each condition and each pulse of the pulse trains. **C)** Peak amplitudes of the C-P2 component for each condition. Data from each participant are represented by gray points and the average of these data points for each condition is represented by colored bars. Numbers 1, 2 and 3 represent the first, second and third pulse of the pulse trains. C-P2 peak amplitudes were not significantly different between the two single pulse conditions (p = 0.16). However, C-P2 peak amplitudes significantly decreased during the second pulses of the pulse trains, regardless of pulse duration (p = 0.01).

C-LEPs were observed in 66.7 % of the participants for the 7 ms and 14 ms single pulse conditions, in 86.7 % of the participants for the 7 ms pulse train condition, and in 76.7 % of the participants for the 14 ms pulse train condition. Average waveforms and topographic maps of the LEPs for each condition are presented in Fig. 5A-B. A positive slow wave was observed between 500 ms and 1500 ms in every condition, corresponding to the range of C-fiber conduction velocity, and consistent with the reaction times and second pain perception reported above (see Fig. 4). Trials with a reaction time < 650 ms (7 % of all trials) were first excluded to assess their influence on the brain responses. Their exclusion had no effect on the observed responses. Therefore, all artifact-free trials were included in the EEG analyses, regardless of the reaction times.

The positive slow wave peak amplitude and latency were measured at the electrode showing maximal activity (Pz). Peak amplitude and latency are reported in Table 1 and Fig. 5C. Peak amplitude and latency were not significantly different between the two single pulse conditions (amplitudes: t(29) = 2.0, p = 0.16, η_p_^2^ = 0.07; latencies: t(29) = 0.22, p = 0.6, η_p_^2^ < 0.01). For the pulse train conditions, peak amplitude and latency were compared between the 7 ms and 14 ms conditions and between the three pulses of the pulse trains with a two-way repeated-measures ANOVA (2 pulse durations × 3 pulses [pulse 1, 2 and 3 of each train (pulse repetition)]). Peak amplitude was not significantly different between the 7 ms and the 14 ms conditions (main effect of pulse duration t: F(1,29) = 0.05, p = 0.8, η_p_^2^ < 0.01), but it was significantly different between the three pulses (main effect of pulse repetition: F(2,58) = 5.1, p = 0.02, η_p_^2^ = 0.15). This repetition effect was not significantly different between the 7 ms and the 14 ms conditions (interaction between pulse duration and pulse repetition: F(2,58) = 1.0, p = 0.4, η_p_^2^ = 0.03). For the 7 ms and 14 ms conditions combined (main effect of pulse repetition), the Tuckey HSD test revealed that the peak amplitude was significantly lower for the second pulse compared with the first pulse (p = 0.01) and third pulse (p = 0.002). No other significant effect was observed (all p’s > 0.05). Peak latency was not significantly different between the 7 ms and the 14 ms conditions (main effect of pulse duration: F(1,29) = 0.04, p = 0.9, η_p_^2^ < 0.01), between the three pulses (main effect of pulse repetition: F(1,29) = 0.5, p = 0.6, η_p_^2^ = 0.02), or between the 7 ms and the 14 ms conditions and the three pulses (interaction between pulse duration and pulse repetition: F(2,58) = 1.8, p = 0.2, η_p_^2^ = 0.06).

**Table 1:** C-P2 peak amplitude (µV) and latency (ms) for each condition (mean ± SD).

**Stimulus type**	**Pulse duration**	**Pulse number**	**Amplitude**	**Latency**
Single pulse	7 ms		5.8 ± 2.4	715.6 ± 203.9
	14 ms		5.3 ± 2.7	735.8 ± 206.3
Pulse train	7 ms	1	3.5 ± 1.7	854.3 ± 213.8
		2	2.7 ± 1.7	836.9 ± 197.5
		3	3.1 ± 1.7	854.1 ± 260.1
	14 ms	1	3.4 ± 1.9	800.8 ± 181.8
		2	2.6 ± 1.6	875.9 ± 216.8
		3	3.5 ± 2.8	886.4 ± 296.7

### 3.4 Event-related spectral perturbations

#### 3.4.1 Event-related synchronization between 2 Hz and 5 Hz and from 500 ms to 1200 ms (ROI 1, delta-theta band oscillations)

**Fig. 6:**
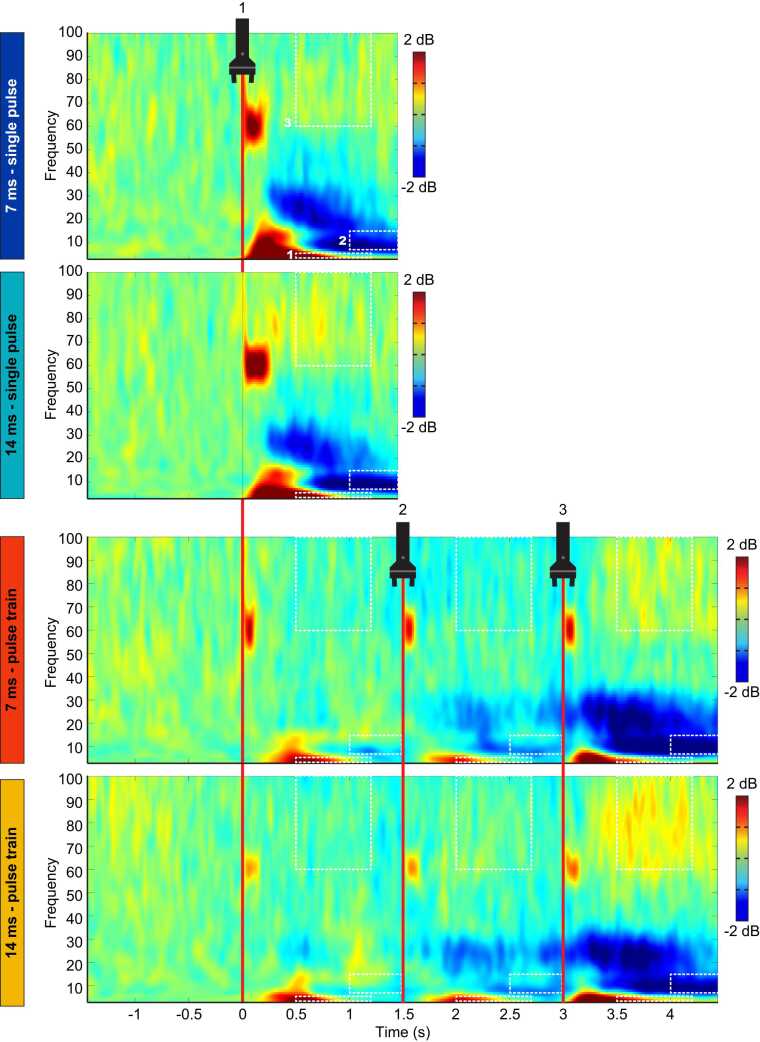
Average time-frequency maps for each condition, time-locked to the onset of laser stimulation. Average time-frequency maps for each condition in each of the three ROIs. Numbers (1, 2 and 3) represent the first, second and third pulse of the pulse trains. Conditions are indicated on the left with the colored rectangles. Regions of interest are highlighted by the dashed-line rectangles and labeled 1, 2 and 3. Note the desynchronization between 10 Hz and 30 Hz and the synchronization between 60 Hz and 100 Hz. See statistical comparisons in Section 3.5 and Fig. 8. Note the stimulation artifact as a power increase at 60 Hz directly after stimulus onset.

**Fig. 7:**
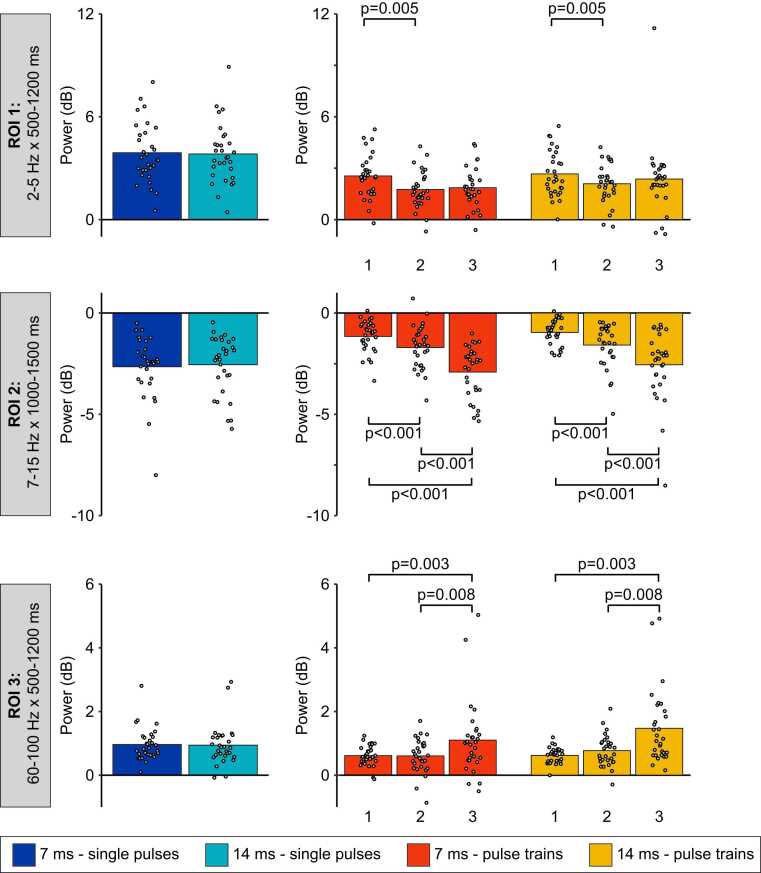
Average ERSP values from the three regions of interest. Data from each participant are represented by gray dots and the average of these data points for each condition is represented by colored bars. Numbers (1, 2 and 3) represent the first, second and third pulse of the pulse trains. For all ROIs, the mean power was not significantly different between the 7 ms and 14 ms single pulse conditions (p > 0.60). For pulse trains in ROI 1, the mean power was not significantly different between the 7 ms and the 14 ms conditions (p = 0.18), but was significantly different between the three pulses (p = 0.01). This effect was not significantly different between the 7 ms and the 14 ms conditions (p = 0.43). For the 7 ms and 14 ms conditions combined (main effect of repetition), the Tuckey HSD test revealed that the mean power significantly decreased during the second pulse compared with the first pulse (p = 0.005), but not compared with the third pulse (p = 0.56). For pulse trains in ROI 2, mean power was not significantly different between the 7 ms and the 14 ms conditions (p = 0.20), but was significantly different between the three pulses (p < 0.001). This effect was not significantly different between the 7 ms and the 14 ms conditions (p = 0.53). For the 7 ms and 14 ms conditions combined (main effect of repetition), the Tuckey HSD test revealed that the desynchronization was significantly greater between the first and second pulses (p < 0.001), between the second and third pulses (p < 0.001), and between the first and third pulses (p < 0.001). For pulse trains in ROI 3, mean power was significantly greater in the 14 ms compared with the 17 ms conditions (p = 0.04). In addition, the mean power significantly increased with pulse repetition (p = 0.001). However, this effect was not significantly different between the 7 ms and the 14 ms conditions (p = 0.14). For the 7 ms and 14 ms conditions combined (main effect of repetition), the Tuckey HSD tests revealed that the mean power significantly increased from the first to the third pulse (p = 0.003), and from the second to the third pulse (p = 0.008), but not from the first to the second pulse (p = 0.43).

**Fig. 8:**
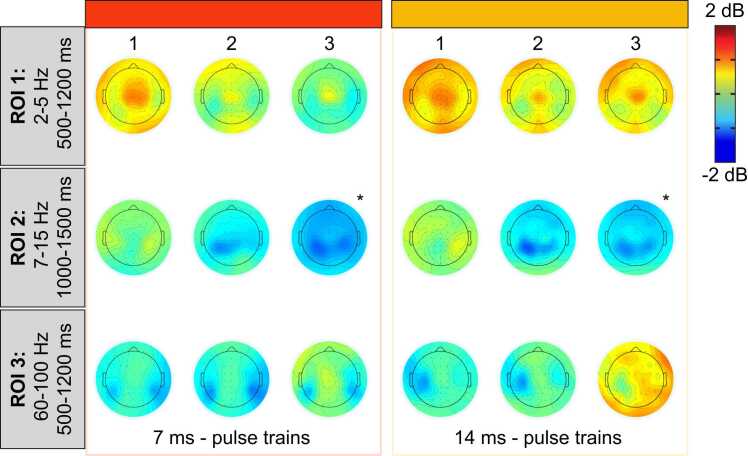
Average topographic maps of the ERSP in the three regions of interest. Average topographic maps of the time-frequency activity in each region of interest for the two pulse train conditions and each pulse of the trains. Numbers 1, 2 and 3 represent the first, second and third pulse of the pulse trains. * Scaling was adjusted from −5 dB to + 5 dB for illustration purposes. See Fig. 7 for statistics.

ERS between 2 Hz and 5 Hz from 500 ms to 1200 ms was not significantly different between the 7 ms and 14 ms single pulse conditions (t(29) = 0.05, p = 0.8, η_p_^2^ < 0.01; see Fig. 6, Fig. 7 and Table 2). For pulse trains, the mean power was not significantly different between the 7 ms and the 14 ms conditions (main effect of pulse duration: F(1,29) = 1.9, p = 0.2, η_p_^2^ = 0.06), but it was significantly different between the three pulses (main effect of pulse repetition: F(2,58) = 5.0, p = 0.01, η_p_^2^ = 0.16) (see Fig. 6, Fig. 7, Fig. 8 and Table 2). This effect was not significantly different between the 7 ms and the 14 ms conditions (interaction between pulse duration and pulse repetition: F(2,58) = 0.8, p = 0.4, η_p_^2^ = 0.03). For the 7 ms and 14 ms conditions combined (main effect of pulse repetition), the Tuckey HSD test revealed that ERS significantly decreased during the second pulse compared with the first pulse (p = 0.005), but not compared with the third pulse (p = 0.6).

**Table 2:** ERSP power (dB) for each condition and region of interest (ROI) (mean ± SD).

**Stimulus type**	**Pulse duration**	**Pulse number**	**ROI 1**	**ROI 2**	**ROI 3**
Single pulse	7 ms		3.9 ± 1.7	−2.7 ± 1.5	1.0 ± 0.5
	14 ms		3.8 ± 1.7	−2.6 ± 1.4	0.9 ± 0.6
Pulse train	7 ms	1	2.5 ± 1.3	−1.2 ± 0.8	0.6 ± 0.3
		2	1.8 ± 1.1	−1.7 ± 1.1	0.6 ± 0.5
		3	1.9 ± 1.2	−2.9 ± 1.3	1.1 ± 1.2
	14 ms	1	2.7 ± 1.3	−1.0 ± 0.6	0.6 ± 0.2
		2	2.1 ± 1.1	−1.6 ± 1.1	0.8 ± 0.5
		3	2.4 ± 2.0	−2.6 ± 1.7	1.5 ± 1.2

#### 3.4.2 Event-related desynchronization between 7 Hz and 15 Hz from 1000 ms to 1500 ms (ROI 2, alpha band oscillations)

ERD between 7 Hz and 15 Hz from 1000 to 1500 ms was not significantly different between the 7 ms and 14 ms single pulse conditions (t(29) = 0.2, p = 0.7, η_p_^2^ < 0.01). For pulse trains, it was not significantly different between the 7 ms and the 14 ms conditions (main effect of pulse duration: F(1,29) = 1.7, p = 0.2, η_p_^2^ = 0.06), but it was significantly different between the three pulses (main effect of pulse repetition: F(2,58) = 56.8, p < 0.001, η_p_^2^ = 0.66). This effect was not significantly different between the 7 ms and the 14 ms conditions (interaction between pulse duration and pulse repetition: F(2,58) = 0.6, p = 0.5, η_p_^2^ = 0.02). For the 7 ms and 14 ms conditions combined (main effect of pulse repetition), the Tuckey HSD test revealed that ERD was significantly greater between the first and second pulses (p < 0.001), between the second and third pulses (p < 0.001), and between the first and third pulses (p < 0.001).

#### 3.4.3 Event-related synchronization between 60 Hz and 100 Hz from 500 ms to 1200 ms (ROI 3, gamma band oscillations)

ERS between 60 Hz and 100 Hz from 500 ms to 1200 ms was not significantly different between the 7 ms and 14 ms single pulse conditions (t(29) = 0.03, p = 0.9, η_p_^2^ < 0.01). For pulse trains, the mean power was significantly greater in the 14 ms compared with the 7 ms conditions (main effect of pulse duration: F(1,29) = 4.6, p = 0.04, η_p_^2^ = 0.14). In addition, the mean power significantly increased with pulse repetition (main effect of pulse repetition: F(2,58) = 11.6, p = 0.001, η_p_^2^ = 0.29). However, this effect was not significantly different between the 7 ms and the 14 ms conditions (interaction between pulse duration and pulse repetition: F(2,58) = 2.2, p = 0.14, η_p_^2^ = 0.07). For the 7 ms and 14 ms conditions combined (main effect of pulse repetition), the Tuckey HSD tests revealed that ERS significantly increased from the first to the third pulse (p = 0.003), and from the second to the third pulse (p = 0.008), but not from the first to the second pulse (p = 0.43).

### 3.5 Exploratory correlation analyses

To explore the association between perception and brain responses, exploratory correlation analyses were conducted with pain ratings and C-LEP peak amplitude, as well as the power of brain oscillations in each ROI. The results revealed no significant effect (all p > 0.46).

## 4 Discussion

The trains of 7 ms pulses elicited C-LEPs in the greatest proportion of participants (86.7 %). C-LEPs were observed as a positive slow wave between 500 ms and 1500 ms post-stimulus but did not show temporal summation. ERD (7–15 Hz) and ERS (60–100 Hz) increased with repeated pulses at 0.67 Hz, consistent with temporal summation of second pain and the underlying C-fiber-related processes. These results provide novel data on C-fiber-related brain responses to painful stimuli and an optimized stimulation protocol for future studies that will investigate nociceptive mechanisms related to the lumbar area.

### 4.1 Electrophysiological characteristics of C-fiber-related brain responses

In the present study, the C-LEPs were observed as a positive slow wave centered at Pz between 500 ms and 1500 ms post-stimulus. This is consistent with the time course of pain perception associated with C-fiber activation and with the conduction velocity of C-fibers [65], [67], [68]. It is also coherent with a previous magnetoencephalographic report that showed a laser-evoked C-fiber-related response observed as a slow wave within the same time window [25]. Other studies have reported C-LEPs as a biphasic potential comprising a negative deflection followed by a positive deflection between 350 ms and 2000 ms [29], [31], [36], [37], [41], [61], [69]. It is not clear if these C-LEPs and the positive slow wave observed in the present study reflect the same or distinct neural processes related to C fibers. The N2-P2 complex reported in previous studies may reflect the detection of the initial C-fiber afferent volley, while the slow wave in the present and previous studies [25] may reflect the perceptual processes underlying the characteristic slow and progressively increasing sensation of second pain, spanning over several hundreds of milliseconds. Accordingly, an earlier N2-P2 complex can be elicited by various salient non-nociceptive stimuli at a latency that corresponds to the detection of the stimulus [70]. In addition, most laser stimuli were perceived as non-painful or around pain threshold in studies that reported a C-fiber-related N2-P2 complex [26], [27], [33], [36], [37], [60], [61], [69], [71], [72], [73]. In contrast, laser stimuli evoked moderate pain when a slow wave was observed (current study and [25]. The response shape may thus vary with the number of activated C fiber afferents. However, in a few studies that reported a N2-P2 complex, laser stimuli were also rated as painful [41], [52], [74], so this remains to be clarified in future studies.

### 4.2 Effect of stimulus repetition on C-fiber-related brain responses

Brain activity associated with second pain evoked by repeated stimuli was characterized in a previous functional magnetic resonance imaging (fMRI) study [75]. However, the temporal resolution of fMRI is insufficient to monitor the pulse-by-pulse responses and the progressive increase in response amplitude [75]. A few studies have used repeated laser stimuli to investigate temporal summation of second pain, but C-fiber-related brain responses were not measured [76], [77], [78], [79]. In the present study, repeated laser stimuli increased the perception of second pain. Yet, the increase in C-LEPs amplitude expected after each pulse was not observed. This lack of temporal summation may result from the constant interstimulus interval between each pulse of the trains. Indeed, findings from previous studies indicate that LEP amplitude decreases significantly when temporal predictability is high [45], [46], [47]. This habituation could explain the significant decrease of the positive slow wave amplitude observed during the second pulse of the train. This decrease was followed by an increased response amplitude for the third pulse. This may reflect competing processes where the interplay between temporal summation and habituation results in a relatively small or no change in response amplitude.

In contrast, time-frequency analyses revealed a significant increase in ERD (7–15 Hz) and ERS (60–100 Hz) with pulse repetition. This effect was not observed for ERS between 2 Hz and 5 Hz from 500 ms to 1200 ms, which corresponds to the C-LEP in the frequency domain [31], [41], [52]. This is consistent with the lack of temporal summation observed for C-LEPs in the present study. The discrepancy between the C-LEPs and C-ERSPs may be explained by the latency jitter of C-fibers [41]. As reported in recent reviews, time-frequency analyses may be better suited to measure EEG activity that is not time-locked or subject to latency jitters [2], [62]. Altogether, this indicates that C-ERSPs are better suited for future investigation on the regulation of temporal summation of second pain.

### 4.3 Future directions

The present results indicate that a protocol of repeated laser heat pulses is the most suitable to detect C-fiber-related brain responses. Laser pulse trains produced distinct Aδ-fiber and C-fiber responses and the C-fiber response was observable with no or a very small Aδ-fiber response. However, although participants ignored the first pain sensation and reported the second pain sensation, we cannot completely rule out that the Aδ-fiber response partially contributed to the C-fiber response and the reported temporal summation of pain. The skin temperature decreases progressively after Nd:YAP laser stimuli [48]. Although reaction times are consistent with the perception of second pain evoked by C fibers, the repeated application of laser heat pulses at a rate of 0.67 Hz may not allow sufficient heat dissipation. In this case, the temporal summation of pain may be caused by increased peripheral afferent activation in addition to wind-up in the dorsal horn of the spinal cord. In future studies, the use of a temperature-controlled feedback laser stimulator would prevent this potential confound.

Laser-evoked brain responses in the present study were produced by back stimulation. It is not clear if the present results apply to hand stimulation, which is the most common stimulation site for LEP studies. The distance between the peripheral afferents and the spinal cord, where windup and temporal summation occur, is longer for the hand and foot compared with the back. Previous studies using contact heat stimulation were designed to investigate the inhibition of temporal summation of second pain by spinal manipulation [80], [81]. In these studies, the lower limb was stimulated because the 3-second back stimuli did not allow distinguishing the second pain from the first pain. The present methods limit this issue because Nd:YAP laser heat pulses produce synchronized nociceptive afferent volleys [82]. Nevertheless, future studies should account for the stimulation site and potential perceptual differences, especially if the stimulation protocol involves repeated stimuli and different body regions.

In the present study, laser stimuli were used to investigate nociceptive processes associated with pain in the lumbar region and temporal summation processes. Although these processes are relevant to low back pain research [17], laser stimuli activate cutaneous A-δ and C fibers, which are less relevant to clinical low back pain than deep tissue nociceptors. However, mechanical stimuli that target deep tissues are not suited for the assessment of temporal summation of C-fiber-related activity, an objective of the present study. Indeed, this requires the repeated application of a noxious stimuli at a relatively high frequency. Therefore, although clinical low back pain is generally driven by nociceptive afferents from deep tissues, cutaneous laser stimuli were the most appropriate for the present study. It should also be noted that laser-evoked potentials are relevant to assess the mechanisms of pain relief by clinical interventions [4], [7].

## 5 Conclusion

Pulse trains of three 7 ms pulses at 0.67 Hz elicited C-LEPs in the greatest proportion of participants (86.7 %), indicating that it is a suitable protocol to measure C-fiber-related brain responses to laser heat pulses applied to the back. These responses included C-LEPs presenting as a slow wave centered at Pz, in addition to ERD (7–15 Hz) and ERS (60–100 Hz), all within a 500 ms to 1500 ms post-stimulus time window, consistent the perception associated with C fiber activation. These results provide novel data on C-fiber-related brain responses to painful stimuli and an optimized stimulation protocol for future studies that will investigate nociceptive mechanisms related to lumbar pain.

## Ethics approval statement

All experimental procedures conformed to the standards set by the latest revision of the Declaration of Helsinki and the guidelines of the International Association for the Study of Pain. The protocol was approved by the Research Ethics Board of Université du Québec à Trois-Rivières and all participants gave written informed consent, acknowledging their right to withdraw from the experiment without prejudice, and received a compensation of $50 for their travel expenses and commitment to the study.

## Funding

This project was funded by the Natural Science and Engineering Research Council of Canada (grant number: 06559), the Fondation Chiropratique du Québec, and the Canadian Foundation for Innovation (grant number: 33731). The contribution of Benjamin Provencher was supported by the 10.13039/100011774Fondation Chiropratique du Québec, the Quebec Bio-Imaging Network and the Fonds de Recherche du Québec – Santé (10.13039/501100000156FRQS).

## CRediT authorship contribution statement

**Provencher Benjamin:** Writing – original draft, Visualization, Validation, Methodology, Investigation, Formal analysis, Data curation, Conceptualization. **Piché Mathieu:** Writing – review & editing, Visualization, Validation, Supervision, Software, Resources, Project administration, Methodology, Investigation, Funding acquisition, Formal analysis, Data curation, Conceptualization.

## Declaration of Competing Interest

The authors declare the following financial interests/personal relationships which may be considered as potential competing interests: Mathieu Piche reports financial support was provided by Natural Sciences and Engineering Research Council of Canada. If there are other authors, they declare that they have no known competing financial interests or personal relationships that could have appeared to influence the work reported in this paper.

## Data Availability

The data that support the findings of this study are available from the corresponding author upon reasonable request.

## References

[1] Plaghki L., Mouraux A. (2005). EEG and laser stimulation as tools for pain research. Curr Opin Investig Drugs.

[2] Verdugo R.J., Matamala J.M., Inui K., Kakigi R., Valls-Solé J., Hansson P. (2022). Review of techniques useful for the assessment of sensory small fiber neuropathies: report from an IFCN expert group. Clin Neurophysiol.

[3] Garcia-Larrea L. (2012). Objective pain diagnostics: clinical neurophysiology. Clin Neurophysiol.

[4] Truini A., Panuccio G., Galeotti F., Maluccio M.R., Sartucci F., Avoli M. (2010). Laser-evoked potentials as a tool for assessing the efficacy of antinociceptive drugs. Eur J Pain.

[5] Provencher B., Northon S., Piché M. (2021). Segmental chiropractic spinal manipulation does not reduce pain amplification and the associated pain-related brain activity in a capsaicin-heat pain model. Front Pain Res.

[6] Provencher B., Northon S., Gevers Montoro C., O’Shaughnessy J., Piché M. (2021). Effects of chiropractic spinal manipulation on laser-evoked pain and brain activity. J Physiol Sci.

[7] Schaffler K., Reeh P., Duan W.R., Best A.E., Othman A.A., Faltynek C.R. (2013). An oral TRPV1 antagonist attenuates laser radiant-heat-evoked potentials and pain ratings from UV(B)-inflamed and normal skin. Br J Clin Pharmacol.

[8] Plaghki L., Mouraux A. (2003). How do we selectively activate skin nociceptors with a high power infrared laser? Physiology and biophysics of laser stimulation. Clin Neurophysiol.

[9] Woolf C.J. (1996). Windup and central sensitization are not equivalent. Pain.

[10] Woolf C.J. (2011). Central sensitization: implications for the diagnosis and treatment of pain. Pain.

[11] Latremoliere A., Woolf C.J. (2009). Central sensitization: a generator of pain hypersensitivity by central neural plasticity. J Pain Off J Am Pain Soc.

[12] Price D.D. (1972). Characteristics of second pain and flexion reflexes indicative of prolonged central summation. Exp Neurol.

[13] Price D.D., Hu J.W., Dubner R., Gracely R.H. (1977). Peripheral suppression of first pain and central summation of second pain evoked by noxious heat pulses. Pain.

[14] Price D.D., Hayes R.L., Ruda M., Dubner R. (1978). Neural representation of cutaneous aftersensations by spinothalamic tract neurons. Fed Proc.

[15] Harte S.E., Harris R.E., Clauw D.J. (2018). The neurobiology of central sensitization. J Appl Biobehav Res.

[16] Georgopoulos V., Akin-Akinyosoye K., Smith S., McWilliams D.F., Hendrick P., Walsh D.A. (2022). An observational study of centrally facilitated pain in individuals with chronic low back pain. Pain Rep.

[17] McPhee M.E., Vaegter H.B., Graven-Nielsen T. (2020). Alterations in pronociceptive and antinociceptive mechanisms in patients with low back pain: a systematic review with meta-analysis. Pain.

[18] Herrero J.F., Laird J.M., Lopez-Garcia J.A. (2000). Wind-up of spinal cord neurones and pain sensation: much ado about something?. Prog Neurobiol.

[19] Price D.D., Staud R., Robinson M.E., Mauderli A.P., Cannon R., Vierck C.J. (2002). Enhanced temporal summation of second pain and its central modulation in fibromyalgia patients. Pain.

[20] Garcia-Larrea L. (2004). Somatosensory volleys and cortical evoked potentials: 'first come, first served'?. Pain.

[21] Bromm B., Neitzel H., Tecklenburg A., Treede R.D. (1983). Evoked cerebral potential correlates of C-fibre activity in man. Neurosci Lett.

[22] Magerl W., Ali Z., Ellrich J., Meyer R.A., Treede R.D. (1999). C- and A delta-fiber components of heat-evoked cerebral potentials in healthy human subjects. Pain.

[23] Bragard D., Chen A.C., Plaghki L. (1996). Direct isolation of ultra-late (C-fibre) evoked brain potentials by CO_2_ laser stimulation of tiny cutaneous surface areas in man. Neurosci Lett.

[24] Opsommer E., Weiss T., Plaghki L., Miltner W.H. (2001). Dipole analysis of ultralate (C-fibres) evoked potentials after laser stimulation of tiny cutaneous surface areas in humans. Neurosci Lett.

[25] Ploner M., Gross J., Timmermann L., Schnitzler A. (2002). Cortical representation of first and second pain sensation in humans. Proc Natl Acad Sci.

[26] Iannetti G.D., Truini A., Romaniello A., Galeotti F., Rizzo C., Manfredi M. (2003). Evidence of a specific spinal pathway for the sense of warmth in humans. J Neurophysiol.

[27] Truini A., Galeotti F., Cruccu G., Garcia-Larrea L. (2007). Inhibition of cortical responses to Adelta inputs by a preceding C-related response: testing the "first come, first served" hypothesis of cortical laser evoked potentials. Pain.

[28] Mouraux A., Plaghki L. (2007). Cortical interactions and integration of nociceptive and non-nociceptive somatosensory inputs in humans. Neuroscience.

[29] Terhaar J., Viola F.C., Franz M., Berger S., Bär K.-J., Weiss T. (2011). Differential processing of laser stimuli by Aδ and C fibres in major depression. Pain.

[30] Churyukanov M., Plaghki L., Legrain V., Mouraux A. (2012). Thermal detection thresholds of Aδ- and C-fibre afferents activated by brief CO_2_ laser pulses applied onto the human hairy skin. PLoS One.

[31] Jankovski A., Plaghki L., Mouraux A. (2013). Reliable EEG responses to the selective activation of C-fibre afferents using a temperature-controlled infrared laser stimulator in conjunction with an adaptive staircase algorithm. Pain.

[32] Naro A., Russo M., Leo A., Rifici C., Pollicino P., Bramanti P. (2015). Cortical responsiveness to nociceptive stimuli in patients with chronic disorders of consciousness: do C-fiber laser evoked potentials have a role?. PLoS One.

[33] Torta D.M., Churyukanov M.V., Plaghki L., Mouraux A. (2015). The effect of heterotopic noxious conditioning stimulation on Aδ-, C- and Aβ-fibre brain responses in humans. Eur J Neurosci.

[34] Azevedo E., Silva A., Martins R., Andersen M.L., Tufik S., Manzano G.M. (2016). Activation of C-fiber nociceptors by low-power diode laser. Arq. De. Neuro-Psiquiatr.

[35] Lefaucheur J.P., Abbas S.A., Lefaucheur-Ménard I., Rouie D., Tebbal D., Bismuth J. (2021). Small nerve fiber selectivity of laser and intraepidermal electrical stimulation: a comparative study between glabrous and hairy skin. Clin Neurophysiol.

[36] Lenoir C., Plaghki L., Mouraux A., van den Broeke E.N. (2018). Quickly responding C-fibre nociceptors contribute to heat hypersensitivity in the area of secondary hyperalgesia. J Physiol.

[37] van den Broeke E.N., Mouraux A. (2014). Enhanced brain responses to C-fiber input in the area of secondary hyperalgesia induced by high-frequency electrical stimulation of the skin. J Neurophysiol.

[38] Mouraux A., Iannetti G.D., Colon E., Nozaradan S., Legrain V., Plaghki L. (2011). Nociceptive steady-state evoked potentials elicited by rapid periodic thermal stimulation of cutaneous nociceptors. J Neurosci.

[39] Qiu Y., Inui K., Wang X., Tran T.D., Kakigi R. (2001). Conduction velocity of the spinothalamic tract in humans as assessed by CO_2_ laser stimulation of C-fibers. Neurosci Lett.

[40] Opsommer E., Masquelier E., Plaghki L. (1999). Determination of nerve conduction velocity of C-fibres in humans from thermal thresholds to contact heat (thermode) and from evoked brain potentials to radiant heat (CO_2_ laser). Clin Neurophysiol.

[41] Hu L., Cai M.M., Xiao P., Luo F., Iannetti G.D. (2014). Human brain responses to concomitant stimulation of Adelta and C nociceptors. J Neurosci Off J Soc Neurosci.

[42] Faul F., Erdfelder E., Lang A.G., Buchner A.G.* (2007). Power 3: a flexible statistical power analysis program for the social, behavioral, and biomedical sciences. Behav Res Methods.

[43] Gupta V., Sharma V.K. (2019). Skin typing: Fitzpatrick grading and others. Clin Dermatol.

[44] Bromm B., Jahnke M.T., Treede R.D. (1984). Responses of human cutaneous afferents to CO_2_ laser stimuli causing pain. Exp Brain Res.

[45] Wang A.L., Mouraux A., Liang M., Iannetti G.D. (2010). Stimulus novelty, and not neural refractoriness, explains the repetition suppression of laser-evoked potentials. J Neurophysiol.

[46] Ronga I., Valentini E., Mouraux A., Iannetti G.D. (2013). Novelty is not enough: laser-evoked potentials are determined by stimulus saliency, not absolute novelty. J Neurophysiol.

[47] Iannetti G.D., Hughes N.P., Lee M.C., Mouraux A. (2008). Determinants of laser-evoked EEG responses: pain perception or stimulus saliency?. J Neurophysiol.

[48] Leandri M., Saturno M., Spadavecchia L., Iannetti G.D., Cruccu G., Truini A. (2006). Measurement of skin temperature after infrared laser stimulation. Clin Neurophysiol.

[49] Northon S., Deldar Z., Piché M. (2021). Cortical interaction of bilateral inputs is similar for noxious and innocuous stimuli but leads to different perceptual effects. Exp Brain Res.

[50] Bialosky J.E., George S.Z., Horn M.E., Price D.D., Staud R., Robinson M.E. (2014). Spinal manipulative therapy-specific changes in pain sensitivity in individuals with low back pain (NCT01168999). J Pain.

[51] Slimani H., Plaghki L., Ptito M., Kupers R. (2016). Pain hypersensitivity in congenital blindness is associated with faster central processing of C-fibre input. Eur J Pain.

[52] Mouraux A., Guérit J.M., Plaghki L. (2003). Non-phase locked electroencephalogram (EEG) responses to CO_2_ laser skin stimulations may reflect central interactions between A∂- and C-fibre afferent volleys. Clin Neurophysiol.

[53] Northon S., Deldar Z., Piche M. (2021). Cortical interaction of bilateral inputs is similar for noxious and innocuous stimuli but leads to different perceptual effects. Exp. Brain Res.

[54] Northon S., Deldar Z., Piche M. (2021). Spinal and cerebral integration of noxious inputs in left-handed individuals. Brain Topogr.

[55] Rustamov N., Northon S., Tessier J., Leblond H., Piché M. (2019). Integration of bilateral nociceptive inputs tunes spinal and cerebral responses. Sci. Rep..

[56] Delorme A., Makeig S. (2004). EEGLAB: an open source toolbox for analysis of single-trial EEG dynamics including independent component analysis. J. Neurosci. Methods.

[57] Rodrigues J., Weiß M., Hewig J., Allen J.J.B. (2021). EPOS: EEG processing open-source scripts. Front Neurosci.

[58] Tabachnick B.G., Fidell L.S. (2013). Using Multivariate Statistics.

[59] Pion-Tonachini L., Kreutz-Delgado K., Makeig S. (2019). ICLabel: An automated electroencephalographic independent component classifier, dataset, and website. NeuroImage.

[60] Hüllemann P., Shao Y.Q., Manthey G., Binder A., Baron R. (2016). Central habituation and distraction alter C-fibre-mediated laser-evoked potential amplitudes. Eur J Pain.

[61] Mouraux A., Guerit J.M., Plaghki L. (2004). Refractoriness cannot explain why C-fiber laser-evoked brain potentials are recorded only if concomitant Adelta-fiber activation is avoided. Pain.

[62] Morales S., Bowers M.E. (2022). Time-frequency analysis methods and their application in developmental EEG data. Dev Cogn Neurosci.

[63] Mouraux A., Iannetti G.D. (2008). Across-trial averaging of event-related EEG responses and beyond. Magn Reson Imaging.

[64] Cohen M.X. (2014).

[65] Barrett K.E., Barman S.M., Brooks H.L., Yuan J.X.J. (2019). Ganong's Review of Medical Physiology.

[66] Ploner M., Sorg C., Gross J. (2017). Brain rhythms of pain. Trends Cogn Sci.

[67] Adriaensen H., Gybels J., Handwerker H.O., Van Hees J. (1983). Response properties of thin myelinated (A-delta) fibers in human skin nerves. J Neurophysiol.

[68] Van Hees J., Gybels J. (1981). C nociceptor activity in human nerve during painful and non painful skin stimulation. J Neurol Neurosurg Psychiatry.

[69] Ammendola E., Tancredi G., Ricci K., Falcicchio G., Valeriani M., de Tommaso M. (2022). Assessment of C fibers evoked potentials in healthy subjects by Nd: YAP laser. Pain Res Manag.

[70] Mouraux A., Iannetti G.D. (2018). The search for pain biomarkers in the human brain. Brain a J Neurol.

[71] De Stefano G., Leone C., Di Pietro G., Esposito N., Falco P., Galosi E. (2022). Unravelling the role of unmyelinated nerve fibres in trigeminal neuralgia with concomitant continuous pain. Clin Neurophysiol.

[72] Truini A., Gerardi M.C., Di Stefano G., La Cesa S., Iannuccelli C., Pepe A. (2015). Hyperexcitability in pain matrices in patients with fibromyalgia. Clin Exp Rheumatol.

[73] Cruccu G., Pennisi E., Truini A., Iannetti G.D., Romaniello A., Le Pera D. (2003). Unmyelinated trigeminal pathways as assessed by laser stimuli in humans. Brain a J Neurol.

[74] Opsommer E., Weiss T., Miltner W.H., Plaghki L. (2001). Scalp topography of ultralate (C-fibres) evoked potentials following thulium YAG laser stimuli to tiny skin surface areas in humans. Clin Neurophysiol.

[75] Staud R., Craggs J.G., Robinson M.E., Perlstein W.M., Price D.D. (2007). Brain activity related to temporal summation of C-fiber evoked pain. Pain.

[76] Stancak A., Alghamdi J., Nurmikko T.J. (2011). Cortical activation changes during repeated laser stimulation: a magnetoencephalographic study. PLoS One.

[77] Ylioja S., Carlson S., Raij T.T., Pertovaara A. (2006). Localization of touch versus heat pain in the human hand: a dissociative effect of temporal parameters on discriminative capacity and decision strategy. Pain.

[78] Nielsen J., Arendt-Nielsen L. (1998). The importance of stimulus configuration for temporal summation of first and second pain to repeated heat stimuli. Eur J Pain.

[79] Svensson P., Beydoun A., Morrow T.J., Casey K.L. (1997). Human intramuscular and cutaneous pain: psychophysical comparisons. Exp Brain Res.

[80] Bialosky J.E., Bishop M.D., Price D.D., Robinson M.E., George S.Z. (2009). The mechanisms of manual therapy in the treatment of musculoskeletal pain: a comprehensive model. Man Ther.

[81] George S.Z., Bishop M.D., Bialosky J.E., Zeppieri G., Robinson M.E. (2006). Immediate effects of spinal manipulation on thermal pain sensitivity: an experimental study. BMC Musculoskelet Disord.

[82] Perchet C., Godinho F., Mazza S., Frot M., Legrain V., Magnin M. (2008). Evoked potentials to nociceptive stimuli delivered by CO_2_ or Nd:YAP lasers. Clin Neurophysiol.

